# 7-Ketolithocholic Acid Exerts Anti-Renal Fibrotic Effects Through FXR-Mediated Inhibition of TGF-β/Smad and Wnt/β-Catenin Pathways

**DOI:** 10.3390/ph19010015

**Published:** 2025-12-21

**Authors:** Qicheng Guo, Lianye Peng, Jingyi Zhang, Junming Hu, Yinyin Wang, Jiali Wei, Zhihao Zhang

**Affiliations:** 1 Key Laboratory of Tropical Biological Resources of Ministry of Education and One Health Institute, School of Pharmaceutical Sciences, Hainan University, Haikou 570228, China; guoqc@hainanu.edu.cn (Q.G.); 23221055000025@hainanu.edu.cn (L.P.); zhangjy@hainanu.edu.cn (J.Z.); hujm@hainanu.edu.cn (J.H.); 2 State Key Laboratory of Natural Medicines, Department of TCMs Pharmaceuticals, School of Traditional Chinese Pharmacy, China Pharmaceutical University, Nanjing 211198, China; yinyin.wang@cpu.edu.cn; 3 Department of Nephrology, Hainan Affiliated Hospital of Hainan Medical University (Hainan General Hospital), Haikou 470100, China

**Keywords:** renal fibrosis, FXR, 7-KLCA, TGF-β/Smad pathway, Wnt/β-catenin pathway

## Abstract

**Background/Objectives:** To explore the protective effects of 7-Ketolithocholic acid (7-KLCA) against renal fibrosis and its mechanism, focusing on its interaction with farnesoid X receptor (FXR). **Methods:** In vitro, TGF-β-induced fibrosis in HK-2/NRK-49F cells and LPS-induced inflammation in HK-2 cells were detected by CCK-8, Western blot, and qPCR. In vivo, unilateral ureteral obstruction (UUO) and adenine (Ade)-induced mouse models were treated with a low/high-dose 7-KLCA or losartan. Renal injury was evaluated via H&E/Masson staining, serum creatinine (SCR), and blood urea nitrogen (BUN) levels. The 7-KLCA-FXR interaction was verified by molecular docking, CETSA, and DARTS. FXR downstream genes and related proteins were measured by WB and qPCR. **Results:** 7-KLCA inhibited the expression of fibrotic proteins (fibronectin, collagen-I) and reduced the LPS-induced release of inflammatory factors (IL-1β, IL-6). In mice, it alleviated renal swelling, collagen deposition, and tubular damage, while lowering serum SCR and BUN levels. Mechanistically, 7-KLCA stably bound to the FXR ligand-binding domain, enhanced its thermal stability and degradation resistance. It upregulated FXR and its downstream genes SHP and FGF15, thereby inhibiting the activation of TGF-β/Smad and Wnt/β-catenin pathways. **Conclusions:** This is the first study to clarify the molecular mechanism through which 7-KLCA targets FXR and dually suppresses the key pro-fibrotic pathways TGF-β/Smad and Wnt/β-catenin, thereby exerting anti-renal fibrosis effects.

## 1. Introduction

Chronic kidney disease (CKD) has emerged as a major global public health challenge, characterized by a progressive loss of kidney function and fibrosis [1], affecting approximately 10% of the general population. Renal fibrosis serves as the common end-stage pathway of all forms of CKD, and during which excessive deposition of extracellular matrix (ECM) disrupts the normal structure of the renal parenchyma, ultimately leading to kidney failure [2]. The occurrence and progression of renal fibrosis are characterized by complex interactions between various cell types and signalling pathways, including epithelial–mesenchymal transition (EMT) in renal tubular epithelial cells, activation of renal interstitial fibroblasts, and infiltration of inflammatory cells [3]. However, there seems to be no available effective treatment for renal fibrosis and end-stage kidney disease apart from dialysis and kidney transplantation currently. Consequently, the urgent identification of innovative therapeutic targets and pharmacological agents is imperative for the better clinical management and treatment of CKD.

In recent years, the downregulation of farnesoid X receptor (FXR)—a key nuclear receptor initially identified for liver metabolism but now recognized as a critical regulator of renal health—has been confirmed to be closely associated with the progression of renal fibrosis [4,5]. A comprehensive review of nuclear receptors in kidney disease highlights that FXR dysfunction disrupts renal tubulointerstitial homeostasis, making it a potential therapeutic target for chronic kidney disease (CKD) [5]. As a member of bile acid receptors, FXR’s role in kidney pathology is further supported by recent evidence; bile acid receptor dysregulation (including FXR) contributes to the development of diabetic nephropathy, a common cause of end-stage renal disease, by promoting inflammation and ECM deposition [6]. Consistent with this, previous studies have shown FXR activation exerts renoprotective effects via bile acid metabolism and anti-inflammatory pathways, significantly alleviating renal fibrosis [5,6]. Furthermore, FXR modulates cellular processes such as proliferation, differentiation, and apoptosis through interactions with various transcription factors, regulating the onset and progression of renal fibrosis [7]. Therefore, FXR and its downstream signalling pathways may represent potential therapeutic targets for renal fibrosis.

As a nuclear receptor FXR serves two critical functions, including maintaining bile acid homeostasis and regulating inflammatory responses. 7-Ketolithocholic acid (7-KLCA), a monohydroxy bile acid derivative with a molecular structure similar to endogenous bile acids, is considered a potential compound for interacting with FXR. In addition, 7-KLCA is closely linked to the enterohepatic circulation and bile acid signalling pathways [8,9]. Although previous studies have confirmed its anti-inflammatory and antioxidant properties, the mechanism between 7-KLCA and renal fibrosis remains unclear. Thus, investigating the linkage between 7-KLCA and FXR could hold promise for uncovering novel mechanisms to ameliorate renal fibrosis and provide new insights and approaches for the treatment of CKD [10,11,12].

In this study, we found that 7-KLCA is capable of inhibiting the activation of the TGF-β/Smad and Wnt/β-catenin signalling pathways, exhibiting the property of reducing the expression of fibrosis-related proteins, and simultaneously alleviating renal tubulointerstitial damage.

## 2. Results

### 2.1. 7-KLCA Ameliorates Renal Fibrosis In Vitro

**Figure 1:**
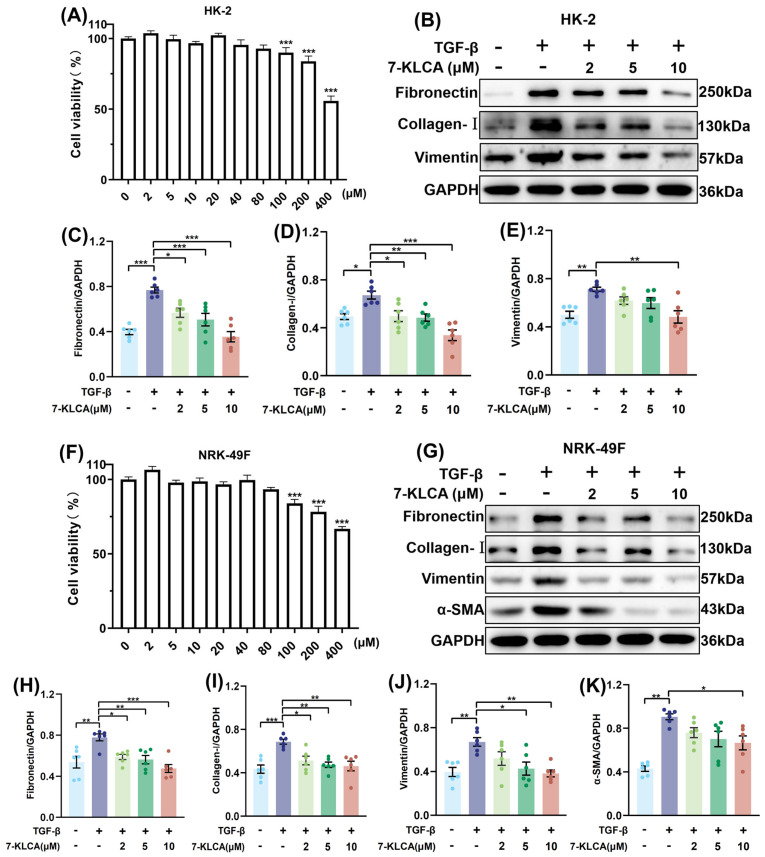
**7-KLCA improves renal fibrosis in vitro.** (**A**) In vitro cell viability test for HK-2 cells treated with 7-KLCA for 24 h (*n* = 6). CCK-8 assay results showed no significant decrease in cell viability when 7-KLCA concentration was ≤100 μM. Therefore, the non-cytotoxic doses of 2 μM, 5 μM, and 10 μM were selected for subsequent TGF-β-induced renal fibrosis experiments. (**B**) The protein expressions of Fibronectin, Collagen-I, Vimentin, and α-SMA in the HK-2 cells from different groups (*n* = 6). (**C**–**E**) Quantitative analysis of (**B**). Data represent the ratio of Fibronectin/Collagen-I/Vimentin band intensity to GAPDH (loading control), normalized to the TGF-β group. (**F**) In vitro cell viability test for NRK-49F cells treated with 7-KLCA for 24 h (*n* = 6). Consistent with the results in HK-2 cells, 7-KLCA did not affect cell viability at concentrations ≤100 μM. The non-toxic doses of 2 μM, 5 μM, and 10 μM were used in subsequent TGF-β-induced fibrosis experiments. (**G**) The protein expressions of Fibronectin, Collagen-I, Vimentin, and α-SMA in the NRK-49F cells from different groups (*n* = 6). (**H**–**K**) Quantitative analysis of (**G**). Data represent the ratio of Fibronectin/Collagen-I/Vimentin/α-SMA band intensity to GAPDH (loading control), normalized to the TGF-β group. * *p* < 0.05, ** *p* < 0.01, *** *p* < 0.001.

We assessed the impact of 7-KLCA on renal cell viability using the CCK-8 assay. There was no significant difference in HK-2 cells when the 7-KLCA concentration was below 100 μM, whereas it decreased significantly once the concentration exceeded 100 μM (Figure 1A). We next established an in vitro renal fibrosis model by stimulating cells with TGF-β and found that in TGF-β-treated HK-2 cells the levels of several pro-fibrotic markers, including fibronectin, collagen-I, and vimentin proteins, diminished in a dose-dependent manner by 7-KLCA treating (Figure 1B–E), indicating that the fibrosis of HK-2 was suppressed. Furthermore, this inference was further corroborated in normal renal fibroblasts of rats NRK-49F. When the 7-KLCA concentration was less than 100 μM, cell viability was not significantly compromised (Figure 1F) and 7-KLCA dose-dependently downregulated the expressions of fibronectin, collagen-I, vimentin, and α-SMA in TGF-β-treated NRK-49F cells (Figure 1G–K). Collectively, these findings demonstrated that 7-KLCA effectively inhibits TGF-β-induced fibrosis in HK-2 and NRK-49F cells.

Following stimulation with LPS in HK-2 cells, the gene expressions of pro-inflammatory cytokines IL-1β, IL-6, and TNF-α were significantly increased. Subsequent to the treatment with 7-KLCA, there was a decline in the relative mRNA expressions of IL-1β, IL-6, and TNF-α in a dose-dependent manner (Figure S1A–C), which suggested that 7-KLCA effectively inhibits LPS-induced inflammation.

### 2.2. 7-KLCA Ameliorates Renal Fibrosis In Vivo

**Figure 2:**
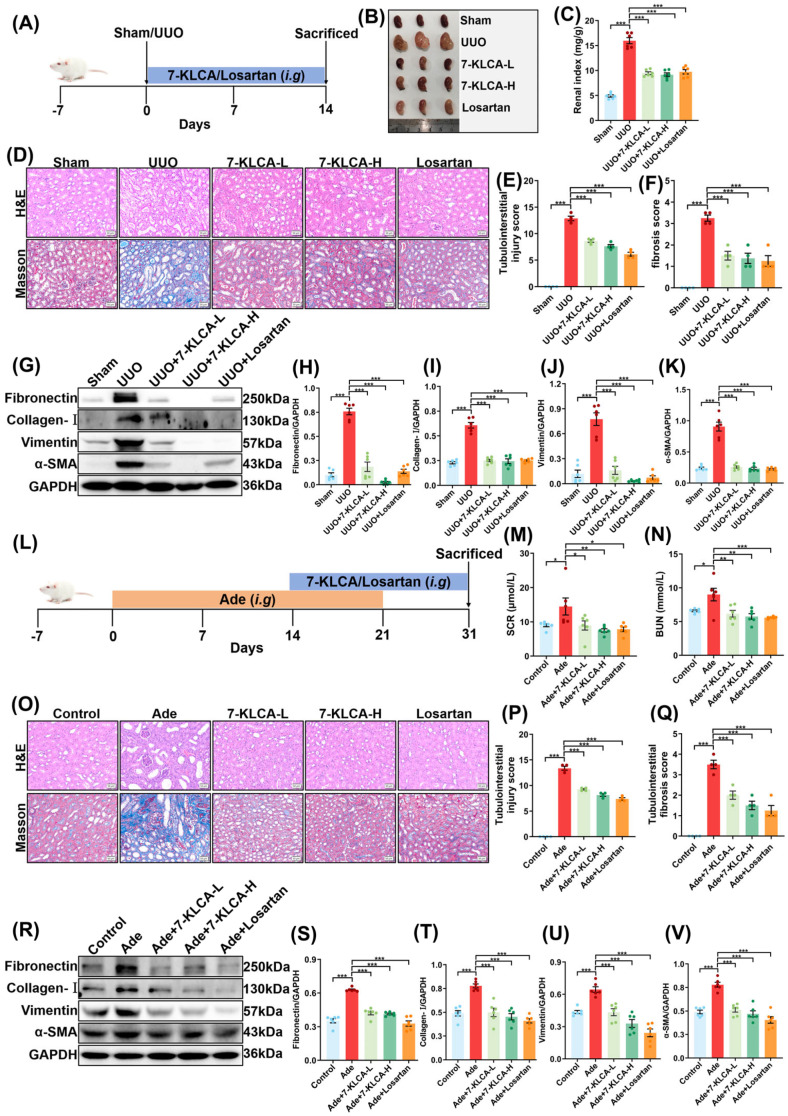
**7-KLCA improves renal fibrosis in vivo.** (**A**) UUO animal experimental programme protocol. (**B**) Picture of left kidneys of mice with different treatments (*n* = 3). (**C**) The renal index (mg/g) was calculated by dividing the wet renal weight by the bodyweight (*n* = 6). (**D**) Representative micrographs of H&E and Masson staining of the left kidneys of mice in different groups (scale bar: 50 μm; magnification: 200×). (**E**,**F**) The histogram of renal injury and renal interstitial fibrosis score according to the pathological results. (**G**) The expressions of Fibronectin, Collagen-I, Vimentin, and α-SMA in the kidneys of mice based on WB analysis (*n* = 6). (**H**–**K**) Quantitative analysis of (**G**). Data represent the ratio of Fibronectin/Collagen-I/Vimentin/α-SMA band intensity to GAPDH (loading control), normalized to the UUO group. (**L**) Ade animal experimental programme protocol. (**M**,**N**) The expression of SCR and BUN in sera of mice (*n* = 6). (**O**) Representative micrographs of H&E and Masson staining of the left kidneys of mice in different groups (scale bar: 50 μm; magnification: 200×). (**P**,**Q**) The histogram of renal injury and renal interstitial fibrosis score according to the pathological results. (**R**) The expressions of Fibronectin, Collagen-I, Vimentin, and α-SMA in the kidneys of mice based on WB analysis (*n* = 6). (**S**–**V**) Quantitative analysis of (**R**). Data represent the ratio of Fibronectin/Collagen-I/Vimentin/α-SMA band intensity to GAPDH (loading control), normalized to the Ade group. Results are mean ± SD of at least six independent experiments. * *p* < 0.05, ** *p* < 0.01, *** *p* < 0.001.

Encouraged by the robust efficacy observed in cell experiment, we proceeded to validate these findings in mice, as illustrated in Figure 2A. Mice in the sham group exhibited normal kidney morphology, while the left kidneys in the UUO group showed significant swelling due to obstruction. Treatment with 7-KLCA and the positive control losartan resulted in a significant improvement in kidney swelling (Figure 2B). Next, the measurement of the renal index revealed that the 7-KLCA-treated group achieved a reduction comparable to the Losartan group. Subsequent to treatment with 7-KLCA, the kidney indices in the 7-KLCA low dose (7-KLCA-L) and 7-KLCA high dose (7-KLCA-H) groups were found to be considerably lower than those observed in the UUO group, and the kidney index in the Losartan group was also found to be significantly reduced (Figure 2C).

The results of the H&E staining showed that the renal tubules in the sham group had clear structures with no obvious interstitial damage. In the UUO group, the renal tubules exhibited widespread dilation, atrophy, and cast formation, with severe interstitial inflammatory cell infiltration. The extent of tubular damage was reduced in the 7-KLCA-L, 7-KLCA-H, and Losartan groups, with improved tubular dilation and atrophy and reduced inflammatory infiltration. Masson staining revealed extensive deposition of collagen fibres in the renal interstitium of the UUO group. After 7-KLCA intervention, collagen fibre deposition was significantly reduced, with similar improvements observed in the Losartan group (Figure 2D–F), which meant a reduction in renal fibrosis. To further explore the specific mechanism, a Western blot experiment was conducted and we found that in comparison with the UUO group the expressions of Fibronectin, Collagen-I, Vimentin, and α-SMA in kidney tissue were significantly reduced in the 7-KLCA-L, 7-KLCA-H, and Losartan groups (Figure 2G–K). In summary, these findings suggest that 7-KLCA has the capacity to attenuate the renal fibrosis in UUO mice.

After investigating the improvement of the UUO model, we explored that of the Ade model, with their experimental flow shown in Figure 2L. Serum biochemical results demonstrated that serum SCR and BUN levels were significantly elevated in the Ade group compared to those in the control group. Serum SCR and BUN levels in the 7-KLCA-L, 7-KLCA-H, and Losartan groups were significantly lower than those in the Ade group (Figure 2M,N). H&E staining revealed that renal tubules exhibited necrosis, detachment, lumen obstruction, and severe interstitial fibrosis in Ade group. In the 7-KLCA-L, 7-KLCA-H, and Losartan groups, renal tubular damage was alleviated, tubular lumen structure tended towards normal, and interstitial fibrosis was reduced. Masson staining revealed extensive collagen fibre deposition in the renal interstitium of the Ade group and, after, 7-KLCA intervention collagen deposition was significantly reduced (Figure 2O–Q). Western blot results demonstrated that the expressions of fibronectin, collagen-I, vimentin, and α-SMA proteins in kidney tissue were significantly reduced in the 7-KLCA-L, 7-KLCA-H, and Losartan groups compared to the Ade group (Figure 2R–V). Taken together these findings suggests that 7-KLCA has the potential to impede the progression of renal fibrosis in the Ade mice.

### 2.3. FXR Expression Was Reduced in Renal Fibrosis

**Figure 3:**
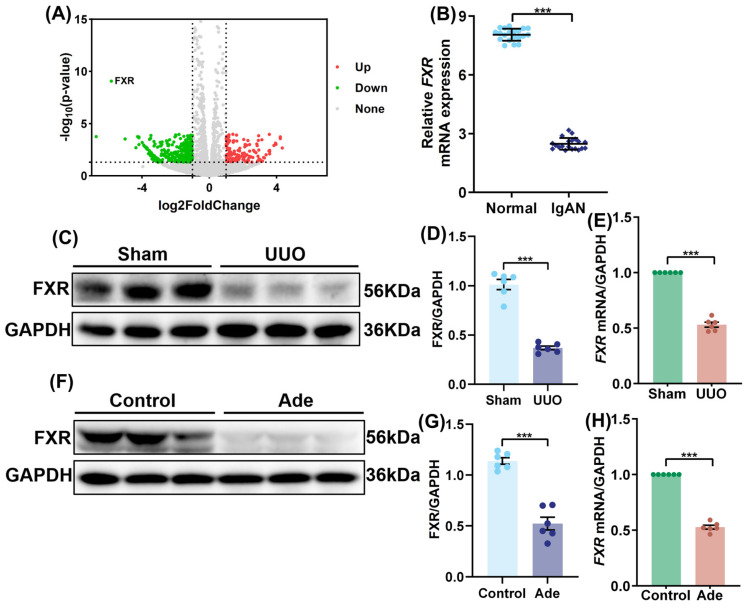
**FXR expression was reduced in renal fibrosis.** (**A**) Transcriptomic analysis of HK-2 cells, the volcano plot showing the differentially expressed genes between control and TGF-β groups (*n* = 3). (**B**) The mRNA levels of FXR based on the GEO database and the differential expression of FXR in the kidneys of 20 healthy individuals and 20 patients with IgA nephropathy compared using the Mann–Whitney Test. (**C**) The expressions of FXR in the kidneys of mice based on WB analysis (*n* = 6). (**D**) Quantitative analysis of (**C**). Data represent the ratio of FXR band intensity to GAPDH (loading control), normalized to the sham group. (**E**) Relative mRNA expression of *FXR* in the kidneys of UUO mice was assessed by qPCR. (**F**) The expressions of FXR in the kidneys of mice based on WB analysis (*n* = 6). (**G**) Quantitative analysis of (**F**). Data represent the ratio of FXR band intensity to GAPDH (loading control), normalized to the Control group. (**H**) Relative mRNA expression of *FXR* in the kidneys of Ade mice was assessed by qPCR. Results are mean ± SD of at least six independent experiments. *** *p* < 0.001.

Transcriptome sequencing results demonstrated that, in HK-2 cells, the FXR gene was significantly downregulated in the TGF-β group in comparison with the normal group (Figure 3A). The analysis of the GEO dataset revealed that the relative mRNA expression of FXR was significantly lower in the kidneys from IgAN patients than that from the healthy individuals (Figure 3B). The results of the Western blot demonstrated a significant reduction in FXR protein expression in the UUO group in comparison with the sham group (Figure 3C,D). Furthermore, quantitative qPCR results confirmed that the mRNA level of FXR mRNA in the UUO group was significantly lower than that in the sham group (Figure 3E). Similarly to the above results, the protein expression of FXR was significantly decreased in the Ade group in comparison to the control group (Figure 3F,G). In addition, the mRNA level of FXR in the Ade group was significantly lower than that in the control group (Figure 3H), which indicates that FXR expression is reduced in renal fibrosis.

### 2.4. 7-KLCA Directly Binds to FXR and Upregulates Its Expression

**Figure 4:**
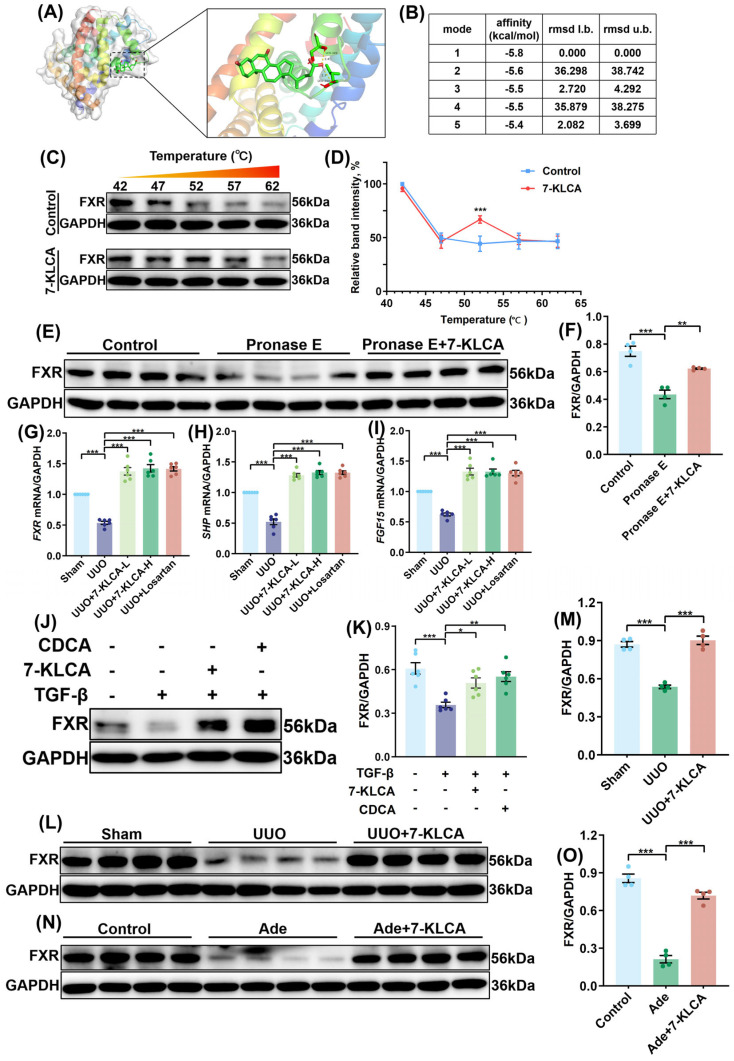
**7-KLCA directly binds to FXR and upregulates its expression.** (**A**) Results of molecular docking between 7-KLCA and FXR. (**B**) Quantitative parameter table for different docking modes. (**C**) WB detection results of cellular thermal shift analysis (*n* = 4). (**D**) Relative intensity quantification curve of the FXR band in (**C**). Data represent the ratio of FXR band intensity to GAPDH (loading control), normalized to the Control group (without 7-KLCA treatment). (**E**) The binding between 7-KLCA and FXR based on drug-affinity-responsive target stability assay (*n* = 4). (**F**) Quantitative analysis of (**E**). Data represent the ratio of FXR band intensity to GAPDH (loading control), normalized to the group without 7-KLCA pre-treatment. (**G**–**I**) Relative mRNA expression of FXR (**G**), SHP (**H**), and FGF15 (**I**) in the kidneys of UUO mice from sham (sham surgery), UUO (model), 7-KLCA-L (20 mg/kg), 7-KLCA-H (40 mg/kg), and Losartan (10 mg/kg) groups was assessed by qPCR. Data are presented as mean ± SD (*n* = 6), with statistical comparisons performed against the UUO model group. (**J**) The protein expressions of FXR in the HK-2 cells from different groups (*n* = 6). (**K**) Quantitative analysis of (**J**). Data represent the ratio of FXR band intensity to GAPDH (loading control), normalized to the TGF-β group. (**L**) The expressions of FXR in the kidneys of mice based on WB analysis (*n* = 4). (**M**) Quantitative analysis of (**L**). Data represent the ratio of FXR band intensity to GAPDH (loading control), normalized to the UUO group. (**N**) The expressions of FXR in the kidneys of mice based on WB analysis (*n* = 4). (**O**) Quantitative analysis of (**N**). Data represent the ratio of FXR band intensity to GAPDH (loading control), normalized to the Ade group. Results are mean ± SD of at least six independent experiments. * *p* < 0.05, ** *p* < 0.01, *** *p* < 0.001.

The molecular docking results demonstrated that 7-KLCA could stably bind to the FXR ligand-binding domain (LBD), with an optimal conformation binding energy of −5.8 kcal/mol (mode 1). It was evident from the analysis of the RMSD value that the binding mode is reliable. Binding-site analysis revealed that 7-KLCA forms hydrogen bonds with FXR’s SER-349 and LEU-344 (Figure 4A,B). The results of the thermal migration assay demonstrated that the control FXR protein underwent gradual degradation with increasing temperature, with relative band intensity decreasing to approximately 30% at 55 °C. Following treatment with 7-KLCA, a significant enhancement in FXR thermal stability was observed, with relative band intensity remaining at approximately 70% at 55 °C. These results indicated that 7-KLCA binding enhances FXR protein thermal stability (Figure 4C,D). The results of the protease protection assay exhibited a near-total degradation of the FXR protein in the Pronase E-treated group. Conversely, the FXR protein retention rate in the 7-KLCA pre-treated group exhibited a significant increase (Figure 4E,F), suggesting that 7-KLCA binding enhances FXR’s resistance to protease hydrolysis and maintains protein stability. The qPCR results demonstrated that compared with the UUO model group, 7-KLCA treatment (both low and high doses) significantly upregulated the mRNA levels of FXR and its downstream target genes SHP and FGF15 in the kidneys of UUO mice, with similar effects observed in the Losartan group (Figure 4G–I). TGF-β induced HK-2 cells showed a significant reduction in FXR protein expression. Treatment with 7-KLCA and CDCA (a FXR agonist) led to a reversal of this reduction (Figure 4J,K). A significant reduction in FXR expression within the kidney tissue of the UUO group, as compared to the sham group, was demonstrated by Western blot analysis, while treatment with 7-KLCA resulted in a significant upregulation of FXR expression (Figure 4L,M). Furthermore, the results of the Western blot indicated a significant decrease in FXR expression in the Ade group compared to the control group, while treatment with 7-KLCA resulted in a significant restoration of FXR expression (Figure 4N,O). In short, we found that 7-KLCA directly binds to the FXR protein in renal cells and upregulates its expression.

### 2.5. Overexpression of FXR Improves Renal Fibrosis and Inhibits TGF-β/Smad Signalling Pathway

**Figure 5:**
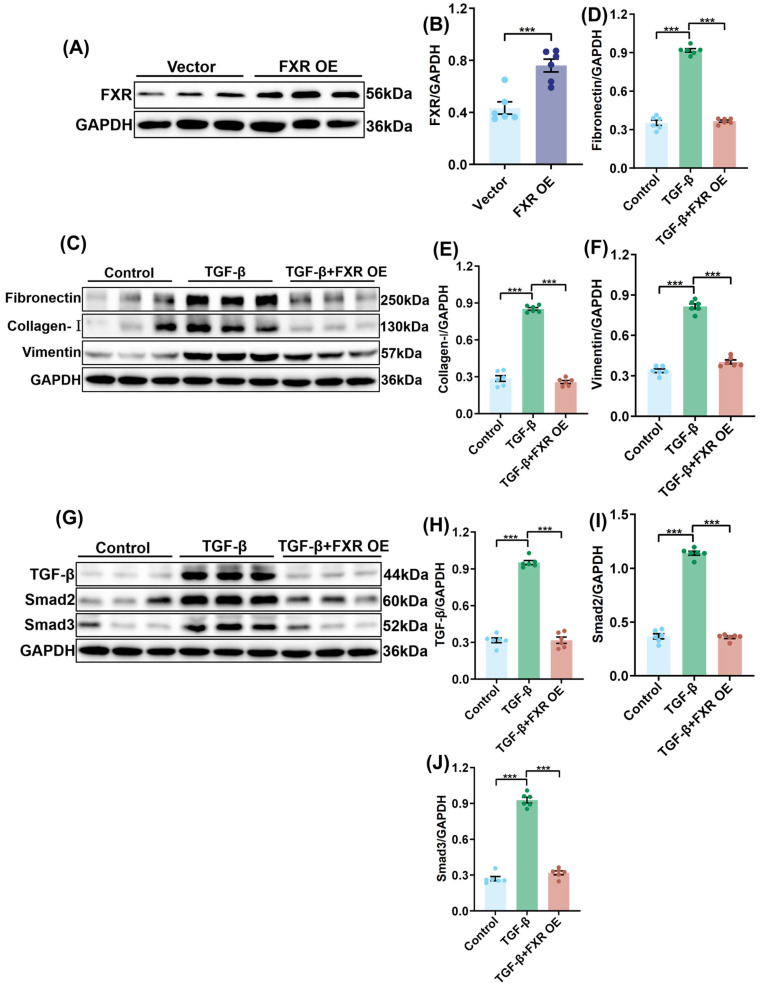
Overexpression of FXR improves renal fibrosis and inhibits TGF-β/Smad signalling pathway. (**A**) The expression levels of FXR protein in HK-2 cells from different groups (*n* = 6). (**B**) Quantitative analysis of (**A**). Data represent the ratio of FXR band intensity to GAPDH (loading control), normalized to the Vector group. (**C**) The protein expressions of Fibronectin, Collagen-I, and Vimentin in the HK-2 cells from different groups (*n* = 6). (**D**–**F**) Quantitative analysis of (**C**). Data represent the ratio of Fibronectin/Collagen-I/Vimentin band intensity to GAPDH (loading control), normalized to the TGF-β group. (**G**) The protein expressions of TGF-β, Smad2, and Smad3 in the HK-2 cells from different groups (*n* = 6). (**H**–**J**) Quantitative analysis of (**G**). Data represent the ratio of TGF-β/Smad2/Smad3 band intensity to GAPDH (loading control), normalized to the TGF-β group. Results are mean ± SD of at least six independent experiments. *** *p* < 0.001.

The protein expression of FXR was significantly increased in the FXR overexpression (FXR OE) group in comparison with the control Vector group (Figure 5A,B), suggesting the successful overexpression of FXR. In comparison with the TGF-β group, the TGF-β + FXR OE group exhibited a marked decrease in the expression of fibronectin, collagen-I, and cimentin proteins (Figure 5C–F). This result demonstrated that FXR overexpression can impede the upregulation of fibrosis-related proteins induced by TGF-β. In addition, in comparison with the TGF-β group, the TGF-β, Smad2, and Smad3 protein expressions were considerably diminished in the TGF-β + FXR OE group (Figure 5G–J). This finding indicates that FXR overexpression can inhibit the activation of the TGF-β/Smad signalling pathway.

### 2.6. 7-KLCA Inhibits TGF-β/Smad and Wnt/β-Catenin Signalling Pathway

**Figure 6:**
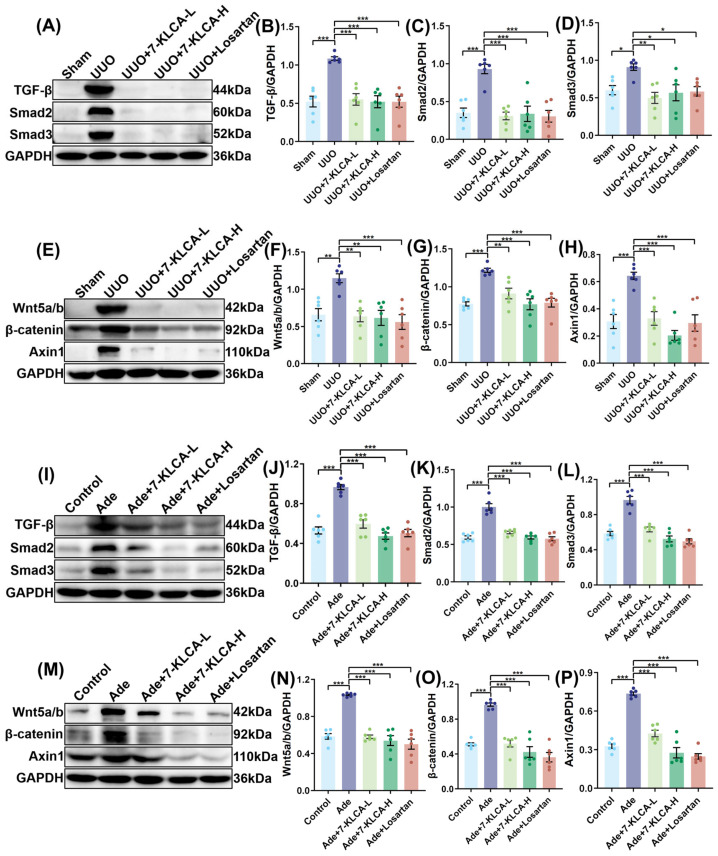
**7-KLCA inhibits TGF-β/Smad and Wnt/β-catenin signalling pathway.** (**A**) The expressions of TGF-β, Smad2, and Smad3 in the kidneys of mice based on WB analysis (*n* = 6). (**B**–**D**) Quantitative analysis of (**A**). Data represent the ratio of TGF-β/Smad2/Smad3 band intensity to GAPDH (loading control), normalized to the UUO group. (**E**) The expressions of Wnt5a/b, β-catenin, and Axin1 in the kidneys of mice based on WB analysis (*n* = 6). (**F**–**H**) Quantitative analysis of (**E**). Data represent the ratio of Wnt5a/b/β-catenin/Axin1 band intensity to GAPDH (loading control), normalized to the UUO group. (**I**) The expressions of TGF-β, Smad2, and Smad3 in the kidneys of mice based on WB analysis (*n* = 6). (**J**–**L**) Quantitative analysis of (**I**). Data represent the ratio of TGF-β/Smad2/Smad3 band intensity to GAPDH (loading control), normalized to the Ade group. (**M**) The expressions of Wnt5a/b, β-catenin, and Axin1 in the kidneys of mice based on WB analysis (*n* = 6). (**N**–**P**) Quantitative analysis of (**M**). Data represent the ratio of Wnt5a/b/β-catenin/Axin1 band intensity to GAPDH (loading control), normalized to the Ade group. Results are mean ± SD of at least six independent experiments. * *p* < 0.05, ** *p* < 0.01, *** *p* < 0.001.

Western blot results demonstrated that the expression of TGF-β, Smad2, and Smad3 proteins was considerably diminished in the 7-KLCA-L, 7-KLCA-H, and Losartan groups in comparison with the UUO group (Figure 6A–D), which suggests that 7-KLCA can inhibit the activation of the TGF-β/Smad pathway in a dose-dependent manner in the UUO model. Likewise, the 7-KLCA intervention led to a substantial downregulation of Wnt5a/b, β-catenin, and Axin1 protein expression in comparison with the UUO group (Figure 6E–H), which indicates that 7-KLCA plays a role in the suppression of Wnt/β-catenin pathway in the UUO model. In addition, Western blot results also demonstrated that TGF-β, Smad2, and Smad3 protein expression was considerably diminished in the 7-KLCA-L, 7-KLCA-H, and Losartan groups in comparison with the Ade group (Figure 6I–L), thereby signifying that 7-KLCA can inhibit TGF-β/Smad pathway in the Ade model in a dose-dependent manner. In a similar manner, the 7-KLCA intervention led to a substantial downregulation of Wnt5a/b, β-catenin, and Axin1 protein expression in comparison with the Ade group (Figure 6M–P). All in all, these results show that 7-KLCA possesses the capacity to impede the activation of the Wnt/β-catenin pathway in the UUO model.

## 3. Discussion

Renal fibrosis is a prevalent pathological process in the progression of chronic kidney disease to end-stage renal disease, characterized by excessive deposition of extracellular matrix and tubulointerstitial inflammation [13]. At present, the range of clinical treatment options for renal fibrosis is limited. While traditional pharmaceuticals such as angiotensin receptor blockers (e.g.; losartan) have demonstrated some efficacy, their long-term utilization is associated with limitations [14]. Consequently, the identification of novel therapeutic targets and drugs is of paramount importance. In this study, through a combination of in vitro and in vivo experiments, we established that 7-KLCA significantly inhibits TGF-β-induced epithelial–mesenchymal transition in HK-2 cells and activation in NRK-49F cells, and reduces the expression of fibrosis-related proteins with no significant effect on cell viability at concentrations below 100 μM. In vivo, UUO and Ade models demonstrated that oral administration of 7-KLCA significantly alleviated kidney index, collagen fibre deposition, and renal tubular damage scores, and reduced serum SCR and BUN levels with effects comparable to those of the positive control losartan. These results suggest that 7-KLCA has potential anti-renal fibrotic effects and its mechanism of action may be related to the inhibition of the TGF-β/Smad and Wnt/β-catenin signalling pathways [15,16].

FXR is a nuclear receptor with dual roles: pivotal regulation of hepatic metabolism and, as a key bile acid receptor, modulation of renal health [17,18]. Recent research on bile acid receptors in diabetic nephropathy emphasize that FXR mediates the crosstalk between bile acid signalling and renal injury—its downregulation during renal fibrosis exacerbates tubulointerstitial inflammation and ECM deposition, while activation reverses these pathological changes [12]. This aligns with our findings: 7-KLCA, a bile acid derivative, binds to FXR and upregulates its expression, thereby inhibiting pro-fibrotic pathways. Notably, FXR’s renoprotective effects via bile acid metabolism and anti-inflammatory pathways have been validated in multiple CKD models, further supporting its therapeutic potential [19,20]. In this study, molecular docking experiments revealed that 7-KLCA forms hydrogen bonds with SER-349 and LEU-344 in the FXR ligand-binding domain, with a binding energy of −5.8 kcal/mol. Thermal shift assays and protease protection assays further validated the direct binding of 7-KLCA to FXR, indicating that 7-KLCA treatment significantly enhances FXR’s thermal stability and resistance to protease degradation [21]. Furthermore, transcriptomic sequencing and analysis of clinical GEO datasets both demonstrated that FXR expression is downregulated in renal fibrosis, while 7-KLCA reverses this trend and upregulates the expression of its downstream target genes SHP and FGF15. These results suggest that the antifibrotic effect of 7-KLCA may be contingent on FXR activation.

The TGF-β/Smad and Wnt/β-catenin signalling pathways represent pivotal pathways in the process of renal fibrosis [22], with their activation promoting the deposition of extracellular matrix and inflammatory responses in the renal tubulointerstitium [22,23]. The present study found that 7-KLCA can inhibit the activation of the TGF-β/Smad signalling pathway in the UUO and Ade models, dose-dependently reducing the expression of TGF-β and Smad2/3 proteins. Furthermore, 7-KLCA has been observed to impede the activation of the Wnt/β-catenin signalling pathway [24,25], leading to a reduction in the expression of Wnt5a/b and β-catenin proteins whilst concurrently reducing the expression of the tumour suppressor factor Axin1. This multi-target regulatory mechanism is hypothesized to stem from FXR-mediated transcriptional regulation, based on existing literature and correlative evidence from our study. Specifically, FXR may competitively bind to DNA response elements with Smad3 or induce its downstream target gene SHP to inhibit β-catenin nuclear translocation—two plausible but unproven mechanisms supported by previous reports on FXR’s crosstalk with pro-fibrotic pathways [26,27,28]. It is important to note that our study did not directly verify these molecular interactions (e.g., FXR-Smad3 physical binding, β-catenin subcellular localization changes, or SHP-β-catenin interaction), and thus these remain speculative working models requiring further validation. Nevertheless, the consistent observations that 7-KLCA directly binds and activates FXR (via molecular docking, CETSA, and DARTS), FXR overexpression mimics 7-KLCA’s anti-fibrotic and pathway-inhibitory effects (Figure 5), and 7-KLCA upregulates FXR downstream genes (SHP, FGF15) while inhibiting both pathways (Figure 4 and Figure 6) strongly supports FXR as a key mediator linking 7-KLCA to the suppression of TGF-β/Smad and Wnt/β-catenin pathways. In comparison with single-pathway inhibitors, the dual-pathway inhibitory properties of 7-KLCA may confer it a competitive advantage in anti-fibrotic therapy as renal fibrosis is driven by the synergistic activation of multiple pro-fibrotic signals.

Clinically, 7-KLCA, as a naturally derived bile acid derivative, offers favourable biocompatibility and dual inhibition of TGF-β/Smad and Wnt/β-catenin pathways—an advantage over single-target agents like losartan. Its ability to reduce SCR, BUN, and collagen deposition may delay CKD progression to ESRD, alleviating reliance on dialysis/transplantation and benefiting patients with comorbid metabolic or liver disorders.

Notably, this study has limitations: animal models (UUO/Ade) do not fully recapitulate human CKD complexity; short-term treatment requires long-term safety/efficacy validation; clinical sample data is lacking to confirm FXR downregulation and 7-KLCA responsiveness in humans; 7-KLCA doses (20/40 mg/kg in mice) need PK/PD analysis for clinical translation; and additional FXR downstream targets contributing to fibrosis warrant exploration. Future research should validate efficacy in patient-derived cells and clinically relevant CKD models, with long-term toxicological and combination therapy studies.

## 4. Materials and Methods

### 4.1. Cell Culture and Treatment

Human renal cortical proximal tubule epithelial cells (HK-2 cells, Chinese Academy of Sciences Stem Cell Bank, Beijing, China) were cultivated in DMEM/F12 medium (C11330500 BT; Gibco, Carlsbad, CA, USA) with the addition of 10% fetal bovine serum (04-001-1ACS, BI) and 1% penicillin-streptomycin (15, 140, 122, Thermo Fisher Scientific, Waltham, MA, USA). Rat renal interstitial fibroblasts (NRK-49F cells, Chinese Academy of Sciences Stem Cell Bank, Beijing, China) were cultured in DMEM medium (01-052-1ACS; BI, Beit Haemek, Israel) with the following supplements: 10% fetal bovine serum (04-001-1ACS, BI, Beit Haemek, Israel) and 1% penicillin-streptomycin (15, 140, 122, Thermo Fisher Scientific, Waltham, MA, USA). All cell lines were cultivated at 37 °C in a 5% CO_2_ environment, and experiments were conducted when cell confluence reached 80–90%. The cells were divided into three distinct groups: a control group, a TGF-β model group (10 ng/mL TGF-β, R&D Systems, Minneapolis, MN, USA), and a TGF-β + 7-KLCA intervention group, with varying concentrations of the latter. The NRK-49F cells were treated in an analogous manner. The cells were collected 48 h after treatment for subsequent analysis.

The in vitro intervention doses of 7-KLCA were set as 2 μM, 5 μM, and 10 μM. The dose selection was based on the following: ① pre-experiment CCK-8 results showed that these doses had no significant inhibitory effect on the viability of HK-2 and NRK-49F cells (Figure 1A,F), avoiding interference from cytotoxicity on experimental results; ② referring to the commonly used effective dose range of bile acid derivatives (e.g., CDCA) in renal fibrosis cell models [10,20], combined with the in vitro activity pre-screening results of 7-KLCA. This dose gradient was determined to evaluate the dose-dependent anti-fibrotic and anti-inflammatory effects of 7-KLCA.

### 4.2. Cell Counts Kit-8 (CCK-8) Assay

The cells were seeded at a density of 1 × 10^4^ cells/well in a 96-well plate and cultured until they adhered to the plate before treatment. The experimental design entailed the establishment of three replicate wells within each group. The CCK-8 reagent was added to the experimental group of 10 μL with this administration occurring 2 h prior to the conclusion of the intervention. Following a 24 h incubation period at 37 °C, the optical density was measured at a wavelength of 450 nm using a microplate reader in order to assess cell viability.

### 4.3. Western Blot Analysis

The Western blot protocol is as follows: for cell samples, lyse with RIPA lysis buffer (containing protease inhibitors) on ice for 30 min; for mouse kidney tissues, add pre-cooled RIPA lysis buffer (containing protease inhibitors) for thorough homogenization, then lyse on ice for 30 min; centrifuge both samples at 12,000 rpm, 4 °C for 15 min to collect supernatants; determine protein concentration by BCA assay, load 30 μg total protein for SDS-PAGE electrophoresis, and transfer to 0.45 μm PVDF membrane; block the membrane with 5% non-fat milk at room temperature for 1 h, incubate with primary antibodies against target proteins (including Fibronectin, Collagen-I, Vimentin, α-SMA, FXR, TGF-β, Smad2/3, Wnt5a/b, β-catenin, etc.) diluted at 1:1000–1:5000 at 4 °C overnight; wash the membrane with TBST 3 times (10 min each), incubate with HRP-conjugated secondary antibody diluted at 1:5000–1:10,000 at room temperature for 1 h; and after washing with TBST 3 times again, develop signals with ECL chemiluminescence kit, analyze band grey values using ImageJ software Version 1.53t, and calculate the relative expression level as the ratio of target protein grey value to internal reference (GAPDH) grey value. The following antibodies were used: anti-collagen I (ab260043, Abcam, Cambridge, UK), anti-fibronectin (ab2413, Abcam, Cambridge, UK), anti-α-SMA (ab124964, Abcam, Cambridge, UK), anti-vimentin (5741, CST, Danvers, MA, USA), anti-NR1H4 (25055-1-AP, Proteintech, Rosemont, IL, USA), anti-GAPDH (HRP-60,004, Proteintech, Rosemont, IL, USA), and HRP-conjugated goat anti-rabbit IgG (#7074 s, CST, Danvers, MA, USA).

### 4.4. mRNA Isolation and qPCR mRNA

Total mRNA was extracted using a High Pure RNA Isolation Kit following the manufacturer’s instructions. Total RNA was reverse transcribed using a HiScript II QRT SuperMix (Version: R223-01; Vazyme Biotech, Nanjing, China) for qPCR in accordance with the manufacturer’s protocol. qPCR was performed on the Step One System, utilizing AceQ qPCR SYBR Green Master Mix. The mRNA levels of target genes were determined by normalizing to the endogenous controls Gapdh. Primer sequences for the genes are detailed in Table 1.

### 4.5. Animal Models

4–6 weeks old ICR male mice, weighing 18–22 g, were purchased from Spelfo (China, Beijing) Biotechnology Co., Ltd. Animal qualification certificate number was SYXK (Qiong) 2023-0031. All research protocols were approved by the Animal Ethics Committee of the School of Pharmacy, Hainan University (HPIACUC2024021). Mice were housed in pathogen-free, well-ventilated cages under a 12 h light/dark cycle, at a room temperature of 25 ± 2 °C, and humidity of 40–60%.

UUO mouse model establishment as previously reported [2]. Mice were administered oral adenine at 180 mg/kg daily for 3 weeks to induce the Ade model. In animal experiments using 7-KLCA, mice were randomly divided into the following groups (using the UUO model as an example; Ade model grouping is similar): Sham group (sham surgery group): only surgical exposure of the ureter was performed without ligation; UUO group (model group): unilateral ureteral ligation was performed to establish a renal fibrosis model; 7-KLCA-L group: 7-KLCA (20 mg/kg) was administered post-UUO surgery; 7-KLCA-H group: 7-KLCA (40 mg/kg) was administered post-UUO surgery; Losartan group: losartan (10 mg/kg) was administered post-UUO surgery. All groups received daily oral administration. 7-KLCA monomer (purity > 98%, batch number: S70983) was purchased from Shanghai Yuanye Biotechnology Co., Ltd., Shanghai, China and losartan (purity > 98%, batch number: HY-175512) was purchased from MedChemexpress Biotechnology Co., Ltd. (Shanghai, China). After the experiment, mice were anesthetized with isoflurane, blood was collected from the right ventricle, and kidneys were harvested and stored at −80 °C until further analysis.

The dose selection was based on the following: ① referring to the effective doses of similar FXR agonists (e.g., obeticholic acid) in mouse kidney disease models [20,26], 20 mg/kg was determined as the basic effective dose; ② the high dose of 40 mg/kg was twice the basic dose to evaluate the dose-dependent effect, and pre-experiments showed that this dose had no obvious acute toxicity in mice (e.g., weight loss, organ damage); ③ it was matched with the clinically equivalent dose of the positive control drug losartan (10 mg/kg) [14] to ensure the comparability of experimental results.

### 4.6. Blood Parameter Measurement and Histology

Blood analysis was performed on an Olympus AU640 automatic analyser. Kidney tissues were fixed in 10% formaldehyde and then embedded in paraffin. H&E Staining: mouse kidney paraffin sections (4–5 μm) were baked at 60 °C for 30 min, dewaxed in xylene I and II for 15 min each, rehydrated in gradient ethanol (100%→95%→80%→70%) for 5 min each, and rinsed with distilled water for 2 min; stained with hematoxylin solution for 3–5 min, rinsed with running water for 1 min, differentiated in 1% hydrochloric acid ethanol for 30 s, and blued in warm water for 5 min; stained with eosin solution for 1–2 min, rinsed with running water for 1 min, then dehydrated in gradient ethanol (70%→80%→95%→100%) for 3 min each, cleared in xylene I and II for 10 min each, and finally mounted with neutral gum and observed under microscope. Masson Staining: mouse kidney paraffin sections were dewaxed and rehydrated as described in HE staining, then stained with hematoxylin solution for 5 min, and rinsed with running water for 2 min; stained with ponceau-acid fuchsin solution for 10 min, gently rinsed with distilled water for 1 min, differentiated in 1% phosphomolybdic acid solution for 5 min, and directly counterstained with aniline blue solution for 5 min; rinsed in 0.5% glacial acetic acid solution for 30 s, rinsed with distilled water for 1 min, dehydrated in gradient ethanol and cleared in xylene (same as HE staining), finally mounted with neutral gum and observed under microscope (collagen fibres: blue-green; cytoplasm: red).

### 4.7. Molecular Docking

As previously mentioned, molecular docking experiments were conducted using AutoDock 4.2.6 to verify the interaction between FXR and 7-KLCA. The mol2 file of the 7-KLCA structure was downloaded from the Traditional Chinese Medicine Systems Pharmacology website and the 3D FXR structure from the Protein Data Bank website. These molecules and structures were then imported into AutoDock 4.2.6 for docking simulations and the results were exported in PDB format. These results were visualized using PyMOL software Version 2.5.0.

### 4.8. Cellular Thermal Shift Analysis (CETSA)

After the cells were well disrupted, 600 μL of PBS was added to each dish, the lysate was collected, and centrifuged to obtain the supernatant; the samples were divided into two parts, with DMSO and 7-KLCA added, respectively, and incubated for 2 h at room temperature. The above cell lysate was then divided into five parts, heated for 3 min at different temperatures (42 °C, 47 °C, 52 °C, 57 °C, and 62 °C), cooled to room temperature and centrifuged to aspirate the supernatant for BCA quantification, and finally subjected to WB analysis.

### 4.9. Drug Affinity Responsive Target Stability (DARTS) Assay

The HK-2 cell lysate was prepared and subsequently diluted to a final protein concentration of 5 μg/mL (determined via Enhanced BCA Protein Assay Kit, consistent with 4.3 Western Blot Analysis for protein quantification). Thereafter, the lysate (with the protein concentration maintained at 5 μg/mL) was incubated at room temperature with 100 µM 7-KLCA (or DMSO as vehicle control) for a period of 1.5 h. Subsequently, Pronase E solution (31.25 ng/mL) was added to the lysate sample (protein:enzyme ratio approximately 800:1), and the mixture was incubated for 10 min at room temperature to initiate limited proteolysis. Following this, the reaction was terminated by adding 5 × SDS-PAGE loading buffer and the protein stability of FXR (the target protein) was analyzed by Western blot Version 1.53t.

### 4.10. Cell Transfection

The pcDNA3.1-NR1H4 and its empty vector pcDNA3.1 were procured from HanHeng Biotechnology (China, Shanghai) Co., Ltd. The cells were detected using LipoFiter 3.0 reagent when they reached 70–80% confluence in a six-well plate in accordance with the instructions provided. Subsequent to the transfection, a series of functional experiments were conducted after 48 h, with the extraction of cellular RNA and total protein. The cells were grouped according to the different plasmids that were transfected.

### 4.11. Statistical Analysis

All statistical analysis is handled uniformly by GraphPad Prism software version 9.0 (GraphPad). Comparisons between two groups were performed using Student’s t-tests. Multigroup comparisons were performed using one-way ANOVA. *p* values less than 0.05 were considered statistically significant.

All quantitative data were subjected to statistical analysis with the mean value of the control/sham group normalized to one; the dispersion of data within each group is represented by error bars (SD), and the *n* value denotes the number of independent experiments or biological samples. This addition serves to explicitly clarify the existence of, and methodology for presenting, dispersion within the control group.

## 5. Conclusions

**Figure 7:**
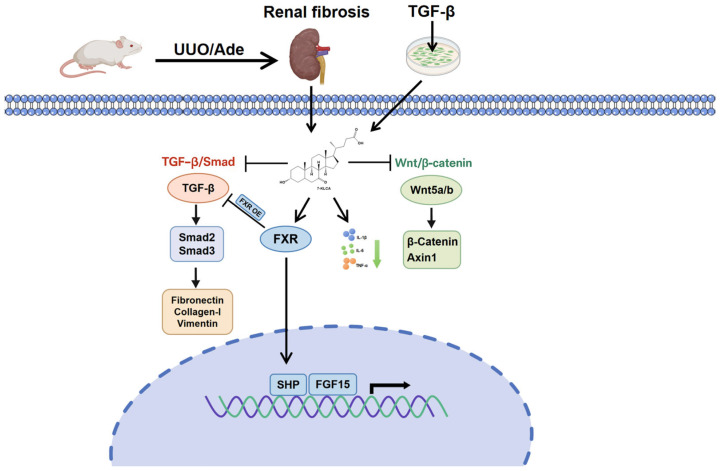
Schematic illustration of the anti-renal fibrotic effect of 7-KLCA.

The present study has revealed the molecular mechanism by which 7-KLCA exerts its anti-renal fibrosis effects by targeting FXR through a combination of in vivo and in vitro experiments. It has demonstrated that 7-KLCA has the capacity to inhibit the activation of the TGF-β/Smad and Wnt/β-catenin signalling pathways and to reduce the expression of fibrosis-related proteins, ultimately leading to alleviated renal tubulointerstitial damage (Figure 7). These results establish a solid theoretical foundation for the development of novel anti-renal fibrosis drugs based on FXR, thereby offering new targets and strategies for the treatment of renal fibrosis.

## Figures and Tables

**Table 1:** qPCR Primer Sequences.

Gene	Forward Primer (5′-3′)	Reverse Primer (3′-5′)
*HK-2 IL-1β*	GCCAGTGAAATGATGGCTTATT	AGGAGCACTTCATCTGTTTAGG
*HK-2 IL-6*	CACTGGTCTTTTGGAGTTTGAG	GGACTTTTGTACTCATCTGCAC
*HK-2 TNF-α*	AGCTGGTGGTGCCATCAGAGG	TGGTAGGAGACGGCGATGCG
*Human GAPDH*	GAAGGTGAAGGTCGGAGTC	GAAGATGGTGATGGGATTTC
*Mouse FXR*	TGTGAGGGCTGCAAAGGTTT	ACATCCCCATCTCTCTGCAC
*Mouse SHP*	TCTGCAGGTCGTCCGACTAT	CAGGCAGTGGCTGTGAGAT
*Mouse FGF15*	TGTACTCCGCTGGTCCCTAT	AGCCCGTATATCTTGCCGTC
*Mouse GAPDH*	CATCAAGAAGGTGGTGAA	AAGTGGAAGAGTGAGTGT

## Data Availability

The original contributions presented in this study are included in the article/Supplementary Materials. Further inquiries can be directed to the corresponding authors.

## Supplementary Materials

The following supporting information can be downloaded at: https://www.mdpi.com/article/10.3390/ph19010015/s1, Figure S1: 7-KLCA inhibits LPS-induced inflammation in HK-2 cells.

## Abbreviations

Ade	Adenine
α-SMA	Alpha smooth muscle actin
BUN	Blood urea nitrogen
CKD	Chronic kidney disease
ECM	Extracellular matrix
FXR	Farnesoid X receptor
FXR OE	FXR overexpression
IL-1β	Interleukin 1 beta
IL6	Interleukin 6
SCR	Serum creatinine
TGF-β	Transforming growth factor β
TNF-α	Tumour necrosis factor alpha
UUO	Unilateral Ureteral Obstruction
7-KLCA	7 Ketolithocholic acid
7-KLCA-L	7 Ketolithocholic acid low dose
7-KLCA-H	7-Ketolithocholic acid high dose
