# Supplementary material for: 7-Ketolithocholic Acid Exerts Anti-Renal Fibrotic Effects Through FXR-Mediated Inhibition of TGF-β/Smad and Wnt/β-Catenin Pathways
Source: Pharmaceuticals (Basel). 2025 Dec 21;19(1):15. doi: 10.3390/ph19010015 (PMC12845293; doi:10.3390/ph19010015)
Supplement: Supplementary file 1 [file pharmaceuticals-19-00015-s001.zip › pharmaceuticals-4013548-supplementary.pdf]

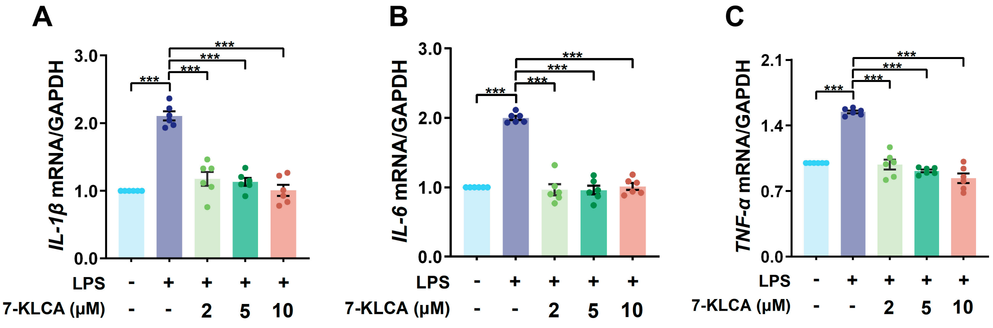

Supplementary Figure S1. 7-KLCA inhibits LPS-induced inflammation in HK-2 cells. Relative mRNA expression of IL-1β (A), IL-6 (B), and TNF-α (C) in HK-2 cells was assessed by qPCR after treatment with 15 ng/mL LPS for 24 h, with or without 7-KLCA intervention (n = 6). Data represent the ratio of target gene mRNA level to GAPDH, normalized to the LPS group. Results are mean ± SD. *$p<0.05$, **$p<0.01$, ***$p<0.001$. This assay demonstrates the broad anti-inflammatory properties of 7-KLCA, which supports its potential anti-fibrotic effect given the crosstalk between inflammation and renal fibrosis.
